# Molecular screening for hemotropic mycoplasmas in captive Barbary sheep (*Ammotragus lervia*) in southern Brazil

**DOI:** 10.14202/vetworld.2017.924-926

**Published:** 2017-08-15

**Authors:** Leonilda C. Santos, Odilon Vidotto, Vivien M. Morikawa, Nelson J. R. Santos, Thállitha S. W. J. Vieira, Ivan R. Barros Filho, Rafael F. C. Vieira, Alexander W. Biondo

**Affiliations:** 1 Department of Sanitary Engineering, Engineering and Exact Sciences Center, Universidade Estadual do Oeste do Paraná, Foz do Iguaçu, PR 85870-650, Brazil; 2 Department of Veterinary Medicine, Universidade Federal do Paraná, Curitiba, PR 80035-050, Brazil; 3 Department of Preventive Veterinary Medicine, Universidade Estadual de Londrina, Londrina, PR 86057-970, Brazil

**Keywords:** aoudads, eperythrozoonosis, hemolytic anemia, hemoplasmas

## Abstract

**Aim:**

This study is part of an active surveillance program for monitoring animal health status in endangered species, and was conducted to screen captive Barbary sheep (*Ammotragus lervia*) for hemoplasma infection.

**Materials and Methods:**

A total of 12 blood samples were collected, DNA extracted and further tested by a pan-hemoplasma polymerase chain reaction protocol.

**Results:**

Animals were clinically healthy and not infested by ectoparasites. Although housekeeping gene DNA was successfully amplified, all the Barbary sheep samples tested negative for *Mycoplasma* sp.

**Conclusion:**

Notwithstanding the negative results, molecular pathogen surveys on Barbary sheep and other exotic wild mammals may provide insights regarding infection of endangered species caused by captivity stress in association with exposure to new pathogens worldwide.

## Introduction

Hemotropic mycoplasmas (hemoplasmas) have been described as pleomorphic and uncultivable bacteria that adhere to the surface of red blood cells in several domestic and wild mammals. They may cause hemolytic anemia and/or subclinical infections [1]. *Mycoplasma ovis* (formerly *Eperythrozoon ovis*) is the main species infecting small ruminants. However, this organism has also been described in other members of the family Bovidae such as the Japanese serow (*Capricornis crispus*) [2], marsh deer (*Blastocerus dichotomus*), red brocket deer (*Mazama americana*), dwarf brocket deer (*Mazama nana*), pampas deer (*Ozotoceros bezoarticus*) [3,4], and reindeer (*Rangifer tarandus*) [5].

Infection by other hemoplasmas have also been described in wild members of the family Bovidae, including *M. ovis*-like in white-tailed deer (*Odocoileus virginianus*) [6], “*Candidatus* Mycoplasma erythrocervae” in marsh deer [4] and sika deer (*Cervus nippon*) [7,8], “*Candidatus* Mycoplasma haemocervae” in sika deer [7,8] and *Mycoplasma* sp. in pampas deer [4], and white-tailed deer [9].

Barbary sheep (*Ammotragus lervia*) or aoudads belong to the subfamily Caprinae, sharing a series of characteristics in common with domestic mammals of the genera *Capra* and *Ovis* [10-12]. Despite being red listed as vulnerable by the International Union for Conservation of Nature [13], no hemoplasma survey has been conducted to date on this animal species.

Accordingly, the aim of this study was to screen captive Barbary sheep at Curitiba Zoo, in southern Brazil, for *Mycoplasma* sp. infection using a previously described pan-hemoplasma polymerase chain reaction (PCR) assay. This study is part of an active surveillance program for monitoring animal health status in endangered species.

## Materials and Methods

### Ethical approval

All samples were collected following ethical methods, as deliberated by the Ethics Committee on the Animal Use of UFPR with protocol number 045/2013.

### Sampling

A total of 12 ethylenediaminetetraacetic acid-blood samples previously surveyed for other pathogens [14] were used in this study. All the samples were stored at − 80°C until molecular procedures were performed.

### DNA extraction and molecular processing

DNA was extracted from 200 µL of blood using a commercially available kit (Illustra Blood Genomic Prep Mini Spin Kit, GE Healthcare, Chalfont St. Giles, UK), in accordance with the manufacturer’s instructions. Negative control purifications using ultrapure water were performed in parallel, to monitor cross contamination. PCR for the housekeeping gene of all mammal species, glyceraldehyde-3-phosphate dehydrogenase, was performed to ensure successful DNA extraction, as previously described [15]. Thereafter, samples were screened by means of conventional pan-hemoplasma PCR targeting the 16S rDNA regions specific for hemoplasmas, using primers that had previously been described by Dieckmann *et al*. [16]. A *M. ovis*-positive goat blood sample was used as positive control. Nuclease-free water and a known uninfected goat sample were used as negative controls.

## Results and Discussion

Although housekeeping gene DNA was successfully amplified, all the Barbary sheep samples tested negative for *Mycoplasma* sp. In this study, the animals were clinically healthy and not infested by ectoparasites, which may explain the negative results. However, we cannot rule out the possibility that these Barbary sheep may have been infected with as yet undescribed hemoplasma species that might not have been amplified by the primer set that was applied.

Among the potential causes of negative results from tests for hemoplasmas, previous reports have suggested that these include healthy animals [17,18]; low parasitemia [19]; sample size and type of population [17]; climatic conditions, presence of the vector and hemoplasma sequestration in tissues [20]; testing conducted during and immediately after antibiotic treatment [21]; and use of inappropriate laboratory tests [19].

To the best of authors’ knowledge, this was the first study to screen Barbary sheep for hemoplasma infection. Active surveillance programs are essential for monitoring the health of endangered species.

## Conclusion

Notwithstanding the negative results, molecular pathogen surveys on Barbary sheep and other exotic wild mammals may provide insights regarding infection of endangered species caused by captivity stress in association with exposure to new pathogens worldwide. Further analysis should be conducted to elucidate whether Barbary sheep could be infected by as yet undescribed hemoplasma species that cannot be amplified through the molecular assays that were applied here.

## Authors’ Contributions

Study design: VMM, RFCV, IRBF, and AWB. Samples collection: VMM, IRBF, and AWB. Laboratory work: LCS, RFCV, OV, NJRS, TSWJV. Manuscript writing was done by LCS, OV, NJRS, TSWJV, VMM, RFCV, IRBF, and AWB. All authors have read and approved the final manuscript.
